# Biomarker testing in oncology – Requirements for organizing external quality assessment programs to improve the performance of laboratory testing: revision of an expert opinion paper on behalf of IQNPath ABSL

**DOI:** 10.1007/s00428-020-02928-z

**Published:** 2020-10-13

**Authors:** K. Dufraing, F. Fenizia, E. Torlakovic, N. Wolstenholme, Z. C. Deans, E. Rouleau, M. Vyberg, S. Parry, E. Schuuring, Elisabeth M. C. Dequeker, on behalf of IQNPath ABSL, N. Normanno, M. H. Cheetham, S. Patton, C. Keppens, K. van Casteren, J. H. van Krieken, J. A. Fairley, M. Grassow-Narlik, K. Jöhrens, J. Pagliuso

**Affiliations:** 1 Biomedical Quality Assurance Research Unit, Department of Public Health and Primary Care, KU Leuven, Kapucijnenvoer 35 blok d, 3000 Leuven, Belgium; 2 Cell Biology and Biotherapy Unit, Istituto Nazionale Tumori “Fondazione G. Pascale”-IRCCS, Naples, Italy; 3 Department of Pathology and Laboratory Medicine, Royal University Hospital, College of Medicine, University of Saskatchewan and Saskatchewan Health Authority, Saskatoon, Saskatchewan Canada; 4 European Molecular Quality Network (EMQN), Manchester Centre for Genomic Medicine, St Mary’s Hospital, Manchester, M13 9WL UK; 5 UK NEQAS for Molecular Genetics, Department of Laboratory Medicine, Royal Infirmary of Edinburgh, Little France Crescent, Edinburgh, EH16 4SA UK; 6 Department of Medical Biology and Pathology, Gustave Roussy, Cancer Genetics Laboratory, Gustave Roussy, Villejuif, France; 7 NordiQC, Institute of Pathology, Aalborg University Hospital, Aalborg, Denmark; 8 UK NEQAS ICC & ISH, University College London Cancer Institute, London, UK; 9 Department of Pathology, University Medical Center Groningen, University of Groningen, Hanzeplein 1, PO Box 30001, 9700 RB Groningen, The Netherlands; 10 Department of Pathology, Radboud University Medical Center, Nijmegen, The Netherlands; 11 Qualitätssicherungs-Initiative Pathologie (QuIP) GmbH, Berlin, Germany; 12 QuIP, Institute of Pathology, Carl Gustav Carus University, Dresden, Germany; 13 RCPA Quality Assurance Programs, St Leonards, NSW Australia

**Keywords:** External quality assessment, Proficiency testing, Predictive biomarkers, Guideline, Molecular pathology, Immunohistochemistry, In situ hybridization, Oncology

## Abstract

In personalized medicine, predictive biomarker testing is the basis for an appropriate choice of therapy for patients with cancer. An important tool for laboratories to ensure accurate results is participation in external quality assurance (EQA) programs. Several providers offer predictive EQA programs for different cancer types, test methods, and sample types. In 2013, a guideline was published on the requirements for organizing high-quality EQA programs in molecular pathology. Now, after six years, steps were taken to further harmonize these EQA programs as an initiative by IQNPath ABSL, an umbrella organization founded by various EQA providers. This revision is based on current knowledge, adds recommendations for programs developed for predictive biomarkers by in situ methodologies (immunohistochemistry and in situ hybridization), and emphasized transparency and an evidence-based approach. In addition, this updated version also has the aim to give an overview of current practices from various EQA providers.

**Electronic supplementary material:**

The online version of this article (10.1007/s00428-020-02928-z) contains supplementary material, which is available to authorized users.

## Introduction

Accurate and reliable biomarker testing is essential to provide optimal personal treatment for patients with cancer. The results of predictive biomarkers often determine which therapy (e.g., chemotherapy, immunotherapy, or targeted therapy) patients receive. Laboratory errors may thus result in wrong or suboptimal treatment decisions and consequently in patient harm. To assure high-quality testing, laboratories should have a quality assurance (QA) system in place and comply with relevant (inter)national standards such as from the International Organization for Standardization (ISO), the College of American Pathologists (CAP), or the Clinical Laboratory Improvement Amendments (CLIA) [1–6]. External quality assurance (EQA), one important component of QA, mostly is a service to diagnostic laboratories by assessment of their testing procedures compared with their peers and/or “designated true value.” Participation in EQA is highly recommended and often mandated [1, 7].

EQA providers distribute testing samples to the laboratories, where the analyses are performed using the same methods as for patient samples. The testing results are then submitted to the EQA provider. In return, the EQA provider performs assessment and analysis of the results and gives tailored feedback on the accuracy of testing and/or reporting. Therefore, the role of EQA is to determine if laboratories provide accurate and complete testing and reporting for patient care [8] using high-quality procedures according to international guidelines [1]. The advantages of participating in EQA programs are widely recognized, which is reflected in the high number of laboratories participating, even where such participation is not mandated [9–25].

The list of current EQA programs is shown in Supplementary Table 1. These programs provide assessments for diagnostic, prognostic, and predictive biomarkers for different cancer types (e.g., lung cancer, colorectal cancer, breast cancer, melanoma), different testing techniques (e.g., polymerase chain reaction (PCR)–based techniques, next-generation sequencing (NGS), immunohistochemistry (IHC), in situ hybridization (ISH)), and different matrices (e.g., fresh, frozen, or formalin-fixed/paraffin-embedded (FFPE) tissue, extracted DNA, cell-free plasma). Since EQA providers are charged with a highly responsible task of assessing the quality of biomarker testing, an international standard exists, ISO/IEC 17043 (Conformity assessment - General requirements for proficiency testing), to provide a framework, requirements, and expectations that need to be met by EQA providers [26]. In addition, the guideline by van Krieken et al. was published in 2013 and is the result of a collaborative effort of several European EQA providers [8]. Under the initiative of the European Society of Pathology, these European EQA providers founded the International Quality Network for Pathology (IQNPath) as an umbrella organization charged with the harmonization of EQA, development and promotion of EQA standards, and provision of a high-level of educational context for EQA [27]. Soon after the inception of IQNPath, several non-European providers (the Canadian Immunohistochemistry Quality Control program (cIQc), the Royal College of Pathologists of Australasia Quality Assurance Programs (RCPAQAP), and CAP) joined the consortium.

The guideline written in 2013 was the start of a process to harmonize the standard of service offered by EQA providers. In retrospect, it has become clear that adhering to some of these recommendations is impossible for a number of EQA schemes with certain designs and objectives, without impacting on the standard of these EQA schemes. For example, if the old guideline had to be followed, the pre- and post-analytical phases would also have to be incorporated any time analytical sensitivity is assessed, while in most cases, this is not the objective of the EQA scheme. To solve these problems, the existing guideline was critically re-assessed as an IQNPath initiative.

Another problem is that the 2013 guideline only focuses on molecular testing, whereas in the current diagnostic routine these are now often associated with IHC and FISH (as a reflex testing strategy). For example, in NSCLC ALK IHC/FISH, ROS1 IHC/FISH and PD-L1 IHC testing are often performed in parallel with *EGFR* gene analysis. Therefore, this update of the guideline will also contain recommendations for IHC/ISH to fulfil the needs of providers who offer schemes for proficiency testing of biomarkers by using various methodologies.

## Methods

Different EQA providers conducting EQA for biomarker testing in oncology shared their knowledge and cumulative experience to update an existing guideline by van Krieken et al. [8]. This update includes recommendations which reflect EQA expert consensus. The whole EQA process from planning the program to communicating the results to participating laboratories is addressed in this paper.

Two surveys (Supplementary Data File 1) were developed and sent to EQA providers (IQNPath members), offering (1) molecular programs: Associazione Italiana di Oncologia Medica (AIOM), European Molecular Genetics Quality Network (EMQN), European Society of Pathology (ESP) EQA, the French national program Gen&Tiss, Quality assurance Initiative Pathology (QuIP), the Royal College of Pathologists of Australia Quality Assurance Programs (RCPAQAP), the Spanish national program (SEAP) and United Kingdom National External Quality Assessment Service (UK NEQAS) for Molecular Genetics/Genomics External Quality Assessment (GenQA) and (2) programs for immunohistochemistry: the Canadian Immunohistochemistry Quality Control program (cIQc), ESP, the Nordic Immunohistochemical Quality Control (NordiQC) and UK NEQAS for Immunocytochemistry & in situ hybridization (ICC & ISH). The survey polled current working strategies of the different EQA providers and expert opinions regarding the existing guideline of van Krieken et al. [8]. Bi-weekly teleconferences were conducted to discuss previous recommendations and to propose additional ones. Final consensus regarding recommendations for organizing EQA programs in oncology was obtained in a face-to-face meeting session during the 7th Meeting on EQA in molecular pathology in Naples, Italy, on May 12th, 2018.

Requirements for laboratory accreditation are outside the scope, although these updated recommendations might help accreditation bodies by informing them of the new concepts and the role of EQA for cancer biomarkers.

## Summary of recommendations

**Table:** 

Ref	Recommendation	New vs carry-over
1. Recommendations for the organization of an EQA program		
1.1	The format of an EQA program should depend on the purpose of the program (integrated approach vs. “test performance characteristic” (TPC)–based approach, see Fig. 1).	New
1.2	The program should be planned and organized by the EQA coordinator considering advice from experts: medical and technical experts and assessors (Supplementary Table 2).	Carry-over
1.3	The time to return results must be pre-defined and monitored.	New
1.4	ISO/IEC 17043 accreditation is strongly recommended.	Carry-over
2. Recommendations for EQA sample selection and validation		
2.1	Samples should be fit for purpose in terms of the investigated TPCs.	New
	Targets should be present in a clinically relevant reportable range, unless pre-determined otherwise.	New
2.2	If possible, sample matrices should be identical to routine samples. Otherwise, substitute matrices could be used (Supplementary Table 3).	New
2.3	Results for challenging cases should be included in the total performance score, unless more than a pre-defined fraction of laboratories had an incorrect result.	New
2.4	The EQA provider is responsible for validation procedures and for the selection of validating laboratories where the validation is conducted. The EQA provider should assess the competence of all laboratories chosen to validate EQA materials.	Carry-over
	Validation of EQA samples is defined as reproducibility of the results in at least two laboratories or by different techniques; one laboratory is always a “designated reference laboratory.” This is the required minimum, but the final validation procedures could be more elaborate and may include other TPCs if deemed necessary by the EQA provider.	New
3. Recommendations for scoring criteria for “pass” vs. “fail”		
3.1	Testing of the pre-analytical phase is generally out of scope of these EQAs.	Carry-over
3.2	For scoring of the analytical phase, a two-tiered system can be used as proposed in Table 1.	Carry-over
	EQA providers should define and monitor “technical malfunctions” and “laboratories with frequent technical malfunctions.”	New
3.3	In schemes with a TPC-focused approach, the following elements should be scored as a minimum: name of the test, sensitivity of the test, and the variants tested. Quality metrics might be scored, depending on the specific methods used for analysis.	New
	In schemes with an integrated approach or TPC-focused schemes where interpretation accuracy is a TPC, the presence and correctness of the interpretation should be scored in relation to the clinical and methodological information. The test interpretation should be written in a general and directive way, unless national guidelines stipulate alternative requirements.	New
4. Recommendations for dealing with poor performance		
4.	EQA providers will report (persistent) poor performers to governmental bodies, if these bodies are available. Where such bodies are not available, it is suggested that EQA providers should perform follow-up studies (e.g., request root cause analysis by the participants) or have to rely on national accreditation bodies for suggestions for improvement and/or could perform additional follow-up studies.	New
5. Communication with participants		
5.	The EQA provider should make efforts for clear communication with laboratories before (e.g., scheme purpose), during (e.g., sample handling), and after result submission (individual results, general report, and appeal phase).	New

## Further clarification of the recommendations

### Organizing an EQA program for oncology biomarkers

#### Scheme with an integrated approach vs “test performance characteristic”–based scheme

**Fig. 1:**
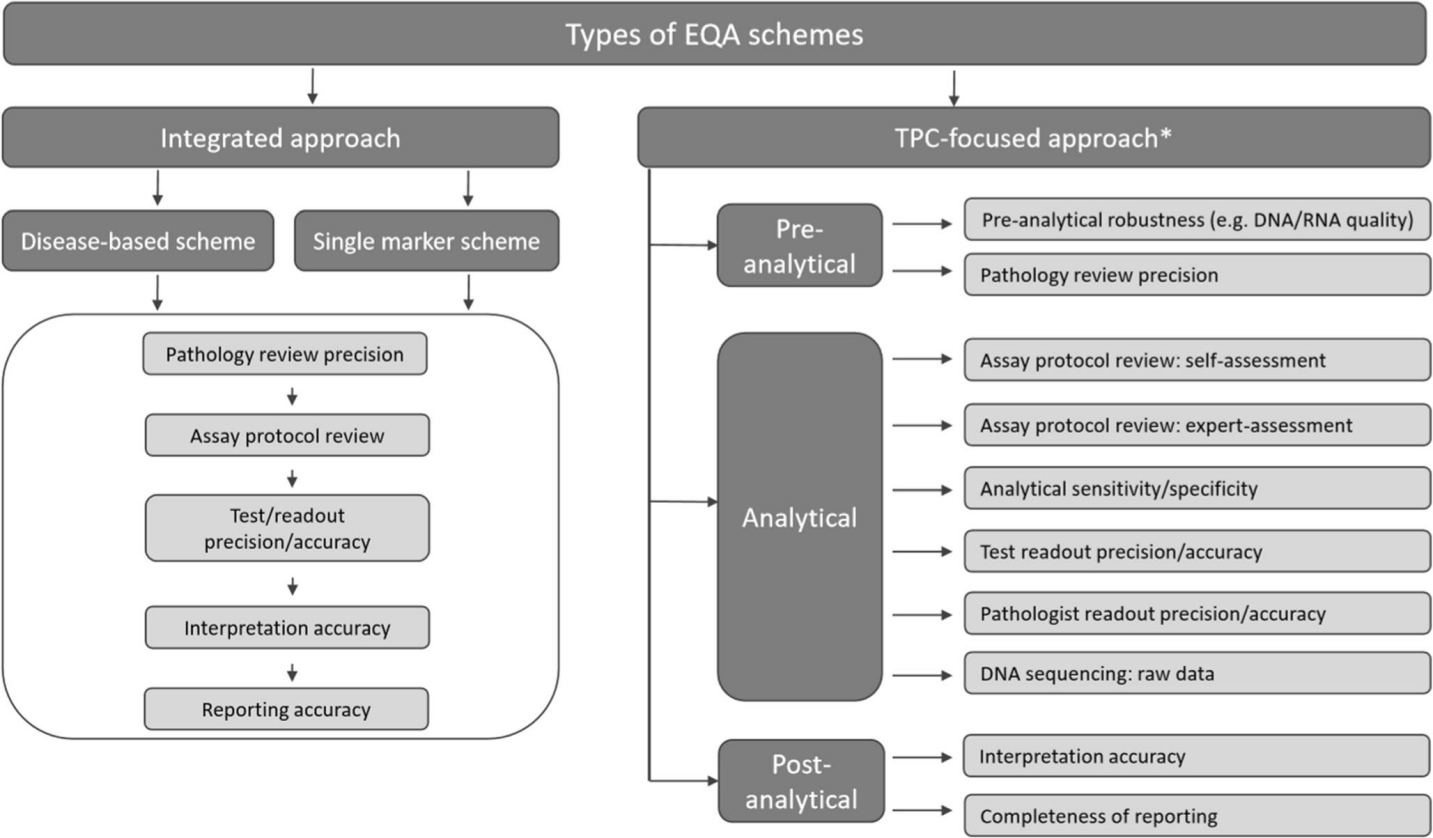
Phases of testing-based approach to EQA as determinant of evidence-based EQA and clinical relevance of EQA results. EQA, external quality assurance; TPCs, test performance characteristics; PPA, positive percent agreement, NPA, negative percent agreement. *Some examples are given; the lists are incomplete

Every laboratory test has three phases: pre-analytical, analytical, and post-analytical. Although the pre-analytical phase includes tissue processing from procurement to the start of assay protocol, this phase is basically outside of the scope of EQA because we assume that diagnostic patient materials selected for EQA in general are uniformly processed. However, on occasion, the pre-analytical phase could be a special subject of the EQA challenge (see recommendations 3.1 and Fig. 1). The analytical phase includes assay protocol and readout. The performance of the assay protocol is often a target of EQA programs. When the readout is generated automatically (e.g., variant calling files for molecular tests), it is not assessed separately from the protocol. However, pathologist’s or (molecular) biologist’s/laboratory scientist readout results (e.g., percent positive cells for IHC or hybridization patterns for ISH), could be assessed for either precision and/or accuracy. The post-analytical phase includes interpretation and reporting of the results.

The design of EQA programs should be fit for purpose from sample selection to result assessment. This purpose must be clearly declared by the provider in advance, to ensure that laboratories are well informed prior to registration.

The EQA providers want to emphasize the importance of distinction between testing phases being evaluated in EQA (Fig. 1). This distinction needs to be declared by the EQA provider. There is an integrated approach which covers the whole testing process (pre-analytical, analytical, and post-analytical), and alternatively, there is the TPC-focused approach aiming at measuring the performance of specific TPCs, typically of 1 testing phase.

The integrated approach is already in practice by several EQA providers. An example is the ESP Lung EQA scheme where participants first need to determine the percentage of neoplastic cells in the sample, followed by testing for molecular alterations and finally submit their result report, including an interpretation of the result. A TPC-based scheme on the other hand can focus on measuring only one or more TPC(s). For example, in the Tissue-i scheme of GenQA, only the pathology review is assessed, and in the ALK IHC program (cIQc), only the diagnostic accuracy is measured.

The selection of TPCs, such as the analytical sensitivity of a test, should be predefined, and sample selection should enable assessment of these TPCs. The level of declared transparency regarding the type of evidence collected is important for understanding the clinical relevance of participation in EQA programs and along that the level of urgency for corrective action.

The EQA providers also want to emphasize the importance of distinction between “pathology review” and “pathologist’s readout” in EQA. “Pathology review” is part of the pre-analytical phase where the pathologist conducts morphological tissue assessment of histology slides with stained tissue sections for the purpose of determining the neoplastic cell percentage, or for determining whether the sample is of acceptable quality. Importantly, tissue slides at different levels throughout the tissue block might be heterogeneous based on molecular/IHC-level content and/or percentage of neoplastic cells, making inter-participant comparison challenging. This type of review may vary depending on the test and sample types and might not be applicable for certain samples (e.g., tissue scrolls, paraffin-embedded cell lines, or liquid biopsies). In contrast, “pathologist’s readout” refers to the analytical phase where pathologist “reads” signals obtained by FISH or IHC protocol on histology slides. For instance, the participating laboratory may return “stained IHC slides” without the pathologist’s readout, where only the IHC protocol performance will be assessed by expert assessment, and where a group of “expert readers” decides whether the IHC slide is positive, negative, or on other characteristics of the staining. However, if the participating laboratory is also asked to submit the pathologist’s readout, this also may become a separate subject of EQA for readout precision and/or accuracy (e.g., evaluation of digital cases in the ESP Lung EQA schemes for FISH and IHC). Also, pathology review and pathologist’s readout should not be confused with the interpretation that is being performed by the pathologist, clinical scientist in molecular pathology, or clinical molecular biologist as a part of the post-analytical phase.

#### EQA experts

According to ISO/IEC 17043:2010 (4.4.1.4), EQA programs need to be organized by an EQA coordinator with access to the necessary technical expertise and experience [26]. The guideline by van Krieken et al. further specified that expertise is to be delivered by a team that includes both a medical and a technical expert. The selection and direct involvement of experts should be appropriate to the purpose of each program as well as an overall purpose of the EQA program. Medical experts should be pathologists if the pathologist is the end user of the test or is performing any component of the test (e.g., pathology review or readout), and it is recommended to involve an oncologist to provide relevant feedback in terms of decision-making for treatment. The EQA providers believe that expert selection does not depend on job titles alone, but on the expertise in the tasks to be performed and related competencies, which is in line with the ISO/IEC 17043:2010 (Supplementary Table 2) [26]. The technical expert in most cases should be a certified molecular biologist (e.g., clinical scientist in molecular pathology) with routine experience for the selected markers in the EQA program. In in situ methods including IHC and FISH, where expertise in morphology is also required, the technical expert is usually a molecular biologist/scientist with significant background education and experience in those specific methods and selected biomarkers. The teamwork between a technical expert and morphology/content expert pathologists is always required for in situ methods. Pathologists could also serve as technical experts, but should have a proven record of technical expertise with the given IHC methodology.

#### Time to return results

The time to return results, being the time between sample receipt by the laboratory and the submission of the test result/reports to the EQA provider, should be determined upfront by the EQA provider based on 2 factors:

- It should not be too short to avoid impact on the processing of patient samples
- It should not be too long so that the assessment and the provision of feedback are not delayed and to ensure adverse clinical impact is not prolonged where relevant.

At the moment, the “time to return results” varies generally between 2 to 8 weeks among the different providers. This time to return results should not be confused with the laboratory’s turnaround time (TAT) for the clinical service, being the time needed to do the test in daily routine. It is not the responsibility of the EQA provider to monitor the laboratory TAT. However, the experts agree that in general laboratories should be able to accommodate the relatively small number of EQA samples in addition to their routine workload.

#### *Accreditation of the EQA program*

The ISO/IEC 17043 standard is used to accredit EQA providers, and it is widely acknowledged that all EQA schemes should obtain accreditation. Furthermore, ISO/IEC 15189:2012 states in a note by item 5.6.3.1 that laboratories should participate in inter-laboratory comparison programs that substantially fulfil the relevant requirements of ISO/IEC 17043 [1]. Currently, most providers in the field of biomarker testing (EMQN, ESP, Gen&Tiss, GenQA, UK NEQAS for ICC & ISH, and RCPAQAP) are already ISO/IEC 17043–accredited or preparing for accreditation (NordiQC, QuIP, and SEAP). Accreditation (or preparation for accreditation) is strongly recommended as it provides a greater assurance that EQA providers have standardized operations and provide transparency regarding their operations and that the minimum quality required is achieved.

### EQA sample selection and sample validation

EQA samples are selected by the EQA coordinator in conjunction with experts and laboratories accredited for ISO15189:2012, under the final responsibility of the EQA coordinator (ISO/IEC 17043:2010 4.4.1.5) [26]. All samples should be selected based on a “fit-for-purpose” approach for EQA (Supplementary Table 3). It is important to adjust the sample type and sample number to the investigated TPCs.

#### Test performance characteristics and their relevance for program design

Various TPCs have been employed for EQA programs, although they are usually not specified. Some examples of TPCs that could be used to assess the performance of the participants with different test phases are shown in Fig. 1. For example, if the purpose of the EQA program is to determine the analytical sensitivity of the method, success in detecting the desired lower limit of detection can be assessed by using either patient material (e.g., human FFPE samples) or other sample types such as cell line–derived FFPE tissue, xenografts, and purified DNA/RNA/peptide control samples/calibrators. When the purpose of the EQA program is to assess readout accuracy or readout precision (e.g., of IHC-stained slides) or interpretation/reporting of the results, glass slides or scans of stained tissue slides could be employed.

Irrespective of the type of the sample, it is recommended that all samples contain targets within clinically relevant reportable ranges [29]. Furthermore, the number of samples with a clinically relevant mutation present, the correct annotation of variants, level of variant allelic frequency (VAF), and sample volume are related to the clinical questions. For example, when the purpose is to determine the overall accuracy of testing for epidermal growth factor receptor (*EGFR*) variants in tissue, this type of program would optimally include a number of samples with different *EGFR* variants in a relevant percentage of neoplastic cells [30]. In contrast, if the purpose of a technical program is assessing the analytical sensitivity for detecting the c.2369C>T; p.(Thr790Met) mutation, a smaller number of samples (≤ 3) with a lower level VAF would suffice. For EQA programs with an integrated approach, variants with different consequences for treatment could be selected.

For IHC-based biomarkers, the clinically relevant reportable range is also critically important. Although new IHC methodologies may increase the analytical sensitivity of IHC methods, the highest achievable analytical sensitivity is not always relevant for clinical practice and diagnostic accuracy [31]. Diagnostic accuracy is addressed through clinical trials or indirect clinical validation. The EQA for any biomarker, including EQA for predictive IHC biomarkers, cannot substitute for clinical validation (which is only achieved in clinical trials) and also rarely provides evidence of indirect clinical validation (which is achieved by following published guidelines), the following types of samples are included in IHC programs which aim at analytical sensitivity and analytical specificity of the IHC protocol: (1) strongly positive sample(s)/cells to confirm that the correct antibody was used; (2) sample(s)/cells with low expression of target protein to determine the sensitivity (low limit of detection); and (3) a negative sample to demonstrate basic analytical specificity [6]. Such prepared samples/cells for testing analytical sensitivity and specificity of IHC biomarkers have been relatively recently coined “immunohistochemistry critical assay performance controls” or iCAPCs [28].

#### Sample matrices

The sample matrix for each tested biomarker should match the matrix of the clinical samples. When not possible, substitute matrices could be used if properly validated for EQA purposes (Supplementary Table 3). For instance, with the large volumes needed for EQA programs, plasma samples cannot be collected from one patient without ethical complications. In these situations, artificial plasma samples spiked with the desired variant at the desirable VAF (in either plasmids or fragmented DNA) are most often used [14, 32]. When low quantities are sufficient, plasma samples from healthy donors can also be used when spiked in the exact same way. Therefore, various factors must be considered in EQA design. For IHC, cell lines or peptide-containing microbeads could also be used either as an add-on to patient tissue samples or independently depending on the purpose of the EQA program [3, 33].

#### Challenging cases

The set of EQA samples should preferably include one or two challenging cases, representing those encountered in real life, for example, samples with low VAFs, rare mutations, and tissue with few neoplastic cells. If more than a pre-defined fraction of laboratories had an incorrect result, the results of this sample should not be included in the performance score and should be marked and reported as “educational case.” “Incorrect” is defined here as a major error that could impact patient outcome such as false positive or false negative results. The authors agree that an EQA program should preferably contain cases with challenging results as these will help participants to critically use EQA results according to ISO15189.

#### Validation of EQA samples

Items to be validated depend on the purpose of EQA programs and should be predefined and related to requirements for routine diagnostics. The EQA providers want to stress the importance of assuring that samples are as homogeneous as possible for the tested parameters and that all participants should receive essentially identical samples (same matrix, same variant, comparable amount of target). Appropriate control steps should be built-in to the process such as evaluating H&E or IHC stained slides from FFPE tissue. It is the responsibility of the EQA coordinator to appropriately select the validating laboratories (also called reference laboratory in ISO/IEC 17043:2010 (A.2)) [26], based on the following selection criteria: (1) they should be a fully equipped pathology (or genetics) laboratory with certified personnel with proven experience in biomarker testing, (2) they should be accredited by ISO/IEC 15189:2012 or ISO/IEC 17020:2012 in certain countries [26, 34], (3) they should use a technology which could demonstrate the pre-set TPC, and (4) they should have passed an EQA program for the same biomarker with the same testing strategy that is applied in the validation, if available.

It was previously determined that samples should be validated by at least two independent laboratories. The requirement remains the same, but the EQA providers now want to nuance that, in the case of patient samples, it is sufficient to test a patient sample once during the routine diagnostic process (if this is performed under the conditions described above for validating laboratories) and once during the validation step. The results between the original testing and from validation laboratories should be identical to include a case in the EQA program. Ideally different methodologies are applied (e.g., NGS and a PCR-based method for molecular schemes, different antibodies for IHC, and different probes for ISH). In case of inconsistency, a third laboratory is needed for confirmation and an explanation for the inconsistency must be given. Samples obtained commercially (e.g., FFPE cell lines, spiked plasma, artificial samples) or externally handed to the EQA provider should be tested by two independent laboratories. The EQA coordinator is responsible for comparing the validation results.

### Scoring criteria for successful vs. unsuccessful participation

The purpose of the EQA program, integrated or TPC-based approach, defines how results are to be scored for “successful” vs. “unsuccessful.” Scoring criteria for each evaluated aspect of the EQA program should be predefined by the EQA provider. Laboratories which have successfully participated could be reported on the website of the EQA provider.

#### Scoring of the test outcome

Scoring criteria for molecular EQA programs remain the same as in the previous guideline to further pursue harmonization between EQA providers (Table 1). For IHC- and ISH-based EQA programs, the purpose could be to assess various TPCs of the analytical phase (Supplementary Table 4), but it is also possible to test for post-analytical factors.

**Table 1:** Proposed scoring criteria for the analytical phase (example given as usually applied by molecular EQA programs)

Scoring criteria	Points
Test outcome	
Target correctly identified	2 points awarded
Target incorrectly identified	0 points awarded
Technical malfunction	0 points awarded (if no valid reason given) 0.5 points awarded (if valid reason given) 1 point awarded (if repeat sample requested)
Nomenclature (If relevant)	
Correct use of HGVS nomenclature [28]	No points deducted
Minor nomenclature error (errors which cannot lead to a misinterpretation of the result), e.g., *EGFR* 2573T>G L858R instead of *EGFR* c.2573T>G p.(Leu858Arg)	No points deducted, but comment given
Major nomenclature error e.g., errors which might cause a misinterpretation of the results; CTG>CGG L858R instead of c.2573T>G p.(Leu858Arg) e.g., only reporting the genomic or protein variant	0.5 points deducted (only once)

HGVS: Human Genome Variation Society (https://varnomen.hgvs.org)

Points should be deducted when laboratories are unable to report a test result due to a technical malfunction. A “technical malfunction” is defined as a random failure of equipment or other parameters where this is not the result of insufficient assay protocol or readout parameters [5]. Since technical malfunctions also happen in routine [35], EQA providers should adapt their score to the reason provided by the laboratory (e.g., laboratories asking for a repeat sample will receive more points than those who reported no reason for failure). EQA providers should encourage participants to report technical malfunctions when they occur. Reporting of technical malfunctions should be accompanied by evidence such as an “error report” from the testing platform or evidence of failure by showing the results with in-house on-slide controls for slide-based technologies (e.g., IHC, ISH). It is also recommended that EQA providers implement a system for monitoring whether the technical malfunction was due to the provided material. In addition, EQA providers could longitudinally follow-up laboratories that frequently report technical malfunctions and provide feedback. What is considered as a “high prevalence of technical malfunctions” is dependent on a number of factors, but mainly on the methodology used and the frequency of EQA participation. Each EQA program should define what requirements are to be met to trigger feedback regarding technical malfunctions to the laboratory.

It is highly recommended that the EQA providers ask the participating laboratories to submit their methods used, including details about which specific method and platform were used and if FDA-approved kits were used. Some participants are using tests that are unable to detect certain alterations at certain VAFs. If testing for these alterations is an absolute requirement for the prescription of a targeted drug or clinically relevant in any other way, participants should be issued the following result: “test not fit for purpose”.

The use of HGVS nomenclature (https://varnomen.hgvs.org/) for reporting alterations should be strongly encouraged by EQA providers as it is essential for correct variant interpretation, reporting, and inclusion in variant databases [36, 37]. For minor nomenclature errors without impact on the interpretation, the correct alternative should be provided to the laboratories. On the other hand, points should be deducted for nomenclature errors leading to potential misinterpretation (e.g., as a false positive or negative result or as a wrong mutation) and potential inappropriate clinical management. In addition, when variants are not correctly reported, they cannot be compared to other studies and/or databases, so the use of HGVS nomenclature is strongly advised.

#### Scoring of the reports

Reporting of the result is a critical element of the testing procedure and all necessary information for correctly interpreting the result should be integrated into the report [4, 38]. Since reports are received by clinicians who may not be experts in the field of biomarker and companion diagnostics, they should be able to interpret the test result in a clear context. It is recommended to have pre-defined scoring criteria which reflect the purpose of the scheme and to provide laboratories with mock information for those elements (Supplementary Table 5). Predefined scoring criteria could be updated to recent developments and approvals of targeted therapies.

In schemes with a TPC-focused approach, providers could check the description of the test method and associated limitations. In general, it must be clearly reported to the clinicians whether (regions with) clinically relevant alterations were not reliably tested. NGS items to be scored on the report are at least the following: name of the gene panel (if commercial panel), sensitivity of the test, minimal coverage and quality metrics including the VAF, coverage, read depth, and reportable range [29]. For non-NGS-based commercial kits, the kit name, sensitivity of the kit, and alterations tested should be scored, and for non-NGS-based non-commercial methods, a brief method description, the sensitivity of the test, and alterations tested. For both in-house developed kits and commercially available kits, the traceability of the included alterations should be verified. For instance, this information can be included as an appendix of the report to increase report readability.

For schemes with an integrated approach, providers should in addition to the elements scored in a TPC-focused approach, score the interpretation of the test outcome in relation to the clinical information that is provided in the test request and the limitations of the test. However, EQA providers realize that in some countries/hospitals an interpretation of the results by pathologists and/or clinical scientist in molecular pathology is not desirable for clinicians. The interpretation must be directive, and therefore, EQA providers should encourage more general interpretation by deducting points or suggesting alternative wording when appropriate. This is particularly the case when new sample types or biomarkers are implemented, for which the interpretation of a result still can be associated with certain pitfalls. For example, for wild-type results for liquid biopsy testing, it is currently not always possible to determine whether a negative test result is a true negative result or the result of a sampling error. It must be noted that participants are not always allowed to submit reports in their mother tongue, and therefore, interpretation errors might result from language issues.

### Dealing with poor performance

To give laboratories specific feedback on their performance, EQA providers should determine whether participation was successful or not. For programs with an integrated approach, the whole testing process should be considered. When doing so, the key question should be, “Will the patient receive incorrect results or advice?” Supplementary Table 6 shows the criteria for successful participation from different providers. Providers agree that laboratories should obtain a minimal score and are not allowed to make major errors to be successful. Of note, most of the time, EQA assessments give only indirect answers to this question by assessing the analytical sensitivity and specificity of the assay, whereas for most assays, there is no established link between its analytical performance and diagnostic test accuracy. Therefore, conclusions based on the analytical performance, where no published evidence exists for its impact on the diagnostic accuracy, need to reflect this limitation and “indirectness of evidence of its impact for patient care.” However, inaccurate reporting can be directly assessed in EQA and it may lead to patient harm. Similarly, EQA programs designed to address diagnostic accuracy may also have more direct evidence of potential for patient harm. Laboratories with continuing suboptimal or poor EQA results should be labelled as “persistent poor performers.” For instance, GenQA defines participants as “persistent poor performers” if they perform poorly in two out of three consecutive EQA rounds [39]. This strategy is also applied in EQA programs for constitutional genetics (e.g., EMQN, CF Network).

EQA providers could (1) report persistent poor performers to governmental bodies, like the National Quality Assessment Advisory Panel (NQAAP) for GenQA, UK NEQAS, and EMQN [39] or (2) if such bodies do not exist, rely on national accreditation bodies. In Belgium for example, there are different Royal Decrees stating that diagnostic tests, including some molecular tests, can only be performed by laboratories accredited by ISO/IEC 15189 and recognized by the Ministry of Public Health [40].

The EQA providers believe that EQA programs should carry both EQA and educational roles. Although the follow-up of actions undertaken after suboptimal outcomes in EQA is not directly within the scope of the EQA provider (ISO/IEC 17043:2010) [26], additional follow-up of these laboratories can still be implemented, if feasible. Several EQA providers have already done this by providing root-cause analysis forms or by doing follow-up studies under a research collaboration [41].

### Communication with participants

The EQA provider should make efforts for clear communication with laboratories. Before the start of each EQA program, providers must communicate the purpose of the program, the tested TPCs, sample types used, the limitations of the EQA design, the scoring criteria, and what type of evidence is generated by the program, the timeline and deadlines, what actions are taken against (persistent) poor performers, and whether laboratories which have successfully participated are reported on the website of the EQA provider or not. This can be communicated via an invitation letter (or follow-up letter), by post, email, via a webpage, or via social media.

When samples are distributed, sufficient information should be given on sample handling, in line with ISO/IEC 17043:2010 (4.6.1.2) [26]. These instructions should be carefully drafted and be specific for the type of material that was sent out.

After analysis, laboratories should submit their results, information about their testing strategies (e.g., type of assay, gene panel, antibody), test limitations, and their written reports, if these are part of the EQA scheme.

After the assessment, results should be communicated individually to each laboratory. Distribution of a table summarizing anonymized scores is also recommended to give laboratories the ability to benchmark themselves compared with peers. In addition, a general report that summarizes all information about the specific EQA challenge/run should be drafted (Table 2). This report has an educational component: scoring criteria are explained, pitfalls highlighted, and average scores given so laboratories can compare their results with colleagues. Often references to relevant publications are provided which can aid in the interpretation of the results. After participating in the EQA program, laboratories should receive a certificate/performance letter clearly indicating whether their participation was successful or not. Laboratories should be offered the opportunity to appeal on their results for a pre-defined period of time. All appeals should be inspected by the program experts and assessors, if feasible. If scores and/or reports are altered following those appeals, this should be communicated to the laboratories. Laboratories which have successfully participated could be reported on the website of the EQA provider, if GDPR and other national or international regulations are considered. On the other hand, laboratories will be notified when authorities and/or national accreditation bodies are notified in case of (persistent) poor performance.

**Table 2:** Elements that should be included in the general EQA report

Element	Further explanation
1. Contact details	Contact details of the EQA provider, the EQA coordinator, and the person(s) of the EQA organization authorizing the report
2. Subcontracted activities	For example, sample validation and preparation
3. Report information	Information should include the issue date, report status, page numbers, report number, name of the EQA program, type of EQA (clinical or technical), and a confidentiality statement.
4. Sample information	A clear description of the selection, validation, and preparation of EQA items used should be given.
5. Marking criteria	Items that were assessed and how scores were given and finally calculated.
6. Participant’s results	Individual and/or aggregate group results. Note: not fit-for-purpose tests can also be listed in the general report
7. Assigned values	These are the correct or expected outcomes of the test.
8. Comments on performance	The EQA coordinator and medical/technical experts give an overview of pitfalls and advice for quality improvement. Results are evaluated and also compared with previous EQA results.
9. Statistics on variation	A descriptive overview showing characteristics of participants, methods, or procedures employed by participants, and the overall success rates should be provided to participants if relevant. Note1: Statistical analysis is not always applicable. Which statistical analyses are applicable is determined by the purpose of the program and selected TPCs. Note 2: Reporting variation in success rate between methods, equipment type, or procedures should be performed with caution. Poor performance is not always directly the result of methodology used, but other factors (human error, pre-analytical errors, reporting errors, etc.) might also play a role. If however a clear problem occurs with one method, reagent, platform, …, the EQA provider should notify the manufacturer and the relevant competent authority in their country of origin according to the IVD regulation [42]. Note 3: It should also be noted that although test validation is not the purpose of EQA programs, laboratories that are still validating their test method may also participate. Depending on the design of the program, their results may contribute to overall evidence for technical validation or indirect clinical validation of the assays.
10. Unusual factors	Situations where unusual factors make evaluation of results and comments on performance impossible. Note: The EQA providers also want to stress that situations where unusual factors make evaluation impossible have not yet occurred in their experience.
11. Conclusions	

The EQA provider can contact laboratories for follow-up studies or to invite them for self-assessment, if applicable. Additionally, good examples of root cause analyses provided by poorly performing laboratories can be shared to improve the understanding and to proactively prevent other laboratories from making the same mistakes. Educational participant workshops may be delivered focusing on educational elements identified through the EQA program. Separately from the reports written by the EQA providers, IQNPath can further educate laboratories about common problems. An example of this is the development of an online digital self-assessment to test readouts for PD-L1 assays (https://cbqareadout.ca/).

## Concluding remarks

External quality assessment is an essential tool that helps laboratories to determine their performance, enables them to compare their performance with their peer group and reference laboratories, and when necessary, improves their biomarker testing practices in oncology. To ensure an objective and high-quality evaluation of performance, practices of different EQA providers further need to be harmonized. With this manuscript, we aimed to update the previous recommendations by van Krieken et al. [8] based on new insights and knowledge while still taking into account current available guidelines and standards such as ISO [1, 26, 36, 43]. In addition, real-life examples were included to inform EQA providers and participating laboratories about the current landscape of EQA for predictive biomarkers. It must be noted that the survey outcomes presented in the Supplementary Data File 1 are a snapshot in time, as these EQA providers continuously implement new EQA schemes. Although EQA results are often shared with accreditation bodies and the success in EQA is often required for laboratory accreditation, requirements for laboratory accreditation are outside the scope of this paper. However, this paper calls for harmonization of EQA programs and it appeals for evidence-based operations and increased transparency of EQA practices. This paper also aims at being helpful to accreditation bodies in their quest of putting proper weight on the meaning of EQA results [44]. It must also be noted that EQA does not replace any components of the regular internal quality control processes.

## Electronic supplementary material

ESM 1 (DOCX 39 kb)

ESM 2 (DOCX 47 kb)

## Abbreviations

AIOM: Associazione Italiana di Oncologia Medica
ASCO: American Society of Clinical Oncology
BRISH: Bright-field in situ hybridization
CAP: College of American Pathologists
cfDNA: Cell-free DNA
cIQc: Canadian Immunohistochemistry Quality Control
CLIA: Clinical Laboratory Improvement Amendments
ctDNA: Circulating tumor DNA
EGFR: Epidermal growth factor receptor
EMQN: European Molecular Genetics Quality Network
EQA: External quality assessment
ESP: European Society of Pathology
FFPE: Formalin-fixed paraffin-embedded
FISH: Fluorescent in situ hybridization
GenQA: Genomics External Quality Assessment
H&E: Hematoxylin and eosin
HER2: Human epidermal growth factor receptor 2
HGVS: Human Genome Variation Society
iCAPs: Immunohistochemistry Critical Assay Performance Controls
IHC: Immunohistochemistry
IQNPath ABSL: International Quality Network for Pathology
ISH: In situ hybridization
ISO: International Organization for Standardization
IEC: International Electrotechnical Commission
IVD: In vitro diagnostics
NGS: Next-generation sequencing
NordiQC: Nordic Immunohistochemical Quality Control
NQAAP: National Quality Assessment Advisory Panel
NSCLC: Non-small cell lung cancer
PT: Proficiency testing
QA: Quality assurance
Quip: Quality assurance Initiative Pathology
RCPAQAP: Royal Collage of Pathologists of Australasia Quality Assurance Programs
SEAP: Sociedad Espanola de Anatomica Patologica
TAT: Turnaround time
TPC: Test performance characteristic
UK NEQAS: United Kingdom National External Quality Assessment Service
VAF: Variant allelic frequency
