# Supplementary material for: Biomarker testing in oncology – Requirements for organizing external quality assessment programs to improve the performance of laboratory testing: revision of an expert opinion paper on behalf of IQNPath ABSL
Source: Virchows Arch. 2020 Oct 13;478(3):553–65. doi: 10.1007/s00428-020-02928-z (PMC7550230; doi:10.1007/s00428-020-02928-z)
Supplement: Supplementary file 1 — (DOCX 39 kb) [file 428_2020_2928_MOESM1_ESM.docx]

**Supplementary table 1** Overview of external quality assessment providers for biomarker testing in oncology

| EQA provider* | | Website | Main publications |
| --- | --- | --- | --- |
| AIOM | Associazione Italiana di Oncologia Medica | www.aiom.it | - Normanno N, Fenizia F, Castiglione F, Barberis M, Taddei GL, Truini M, De Rosa G, Pinto C, Marchetti A (2017) [External quality assessment for EGFR mutations in Italy: improvements in performances over the time.](https://www.ncbi.nlm.nih.gov/pubmed/29181190) ESMO Open 2(2):e000160. - Normanno N, Pinto C, Castiglione F, Fenizia F, Barberis M, Marchetti A, Fontanini G, De Rosa G, Taddei G (2015) The Italian external quality assessment for RAS testing in colorectal carcinoma identifies methods-related inter-laboratory differences. J Transl Med 13:287. |
| EMQN | European Molecular Genetics Quality Network | www.emqn.org | - Ellison G, Wallace A, Kohlmann A, Patton S (2017) A comparative study of germline BRCA1and BRCA2 mutation screening methods in use in 20 European clinical diagnostic laboratories. Br J Cancer 117:710-716. - Haselmann V, Ahmad-Nejad P, Geilenkeuser WJ, Duda A, Gabor M, Eichner R, Patton S, Neumaier M (2018) [Results of the first external quality assessment scheme (EQA) for isolation and analysis of circulating tumour DNA (ctDNA).](https://www.ncbi.nlm.nih.gov/pubmed/28841569) Clin Chem Lab Med 56(2):220-228. |
| ESP EQA | European Society of Pathology External Quality Assessment | lung.eqascheme.org kras.eqascheme.org | - Keppens C, Tack V, Hart N', Tembuyser L, Ryska A, Pauwels P, Zwaenepoel K, Schuuring E, Cabillic F, Tornillo L, Warth A, Weichert W, Dequeker E (2018) A stitch in time saves nine: external quality assessment rounds demonstrate improved quality of biomarker analysis in lung cancer. Oncotarget 9(29):20524-20538. - Tack V, Ligtenberg MJ, Tembuyser L, Normanno N, Vander Borght S, Han van Krieken J, Dequeker EM (2015) [External quality assessment unravels interlaboratory differences in quality of RAS testing for anti-EGFR therapy in colorectal cancer.](https://www.ncbi.nlm.nih.gov/pubmed/25657200) Oncologist 20(3):257-62. |
| Gen&Tiss | French national EQA program | www.genetiss.org | - Dequeker EM, Keppens C, Egele C, Delen S, Lamy A, Lemoine A, Sabourin JC, Andrieu C, Ligtenberg M, Fetique D, Tops B, Descarpentries C, Blons H, Denoux Y, Aube C, Penault-Llorca F, Hofman P, Leroy K, Le Marechal C, Doucet L, Duranton-Tanneur V, Pedeutour F, Soubeyran I, Côté JF, Emile JF, Vignaud JM, Monhoven N, Haddad V, Laurent-Puig P, van Krieken H, Nowak F, Lonchamp E, Bellocq JP, Rouleau E (2016) [Three Rounds of External Quality Assessment in France to Evaluate the Performance of 28 Platforms for Multiparametric Molecular Testing in Metastatic Colorectal and Non-Small Cell Lung Cancer.](https://www.ncbi.nlm.nih.gov/pubmed/26752307) J Mol Diagn 18(2):205-14. - Lhermitte B, Egele C, Weingertner N, Ambrosetti D, Dadone B, Kubiniek V, Burel-Vandenbos F, Coyne J, Michiels JF, Chenard MP, Rouleau E, Sabourin JC, Bellocq JP (2017) [Adequately defining tumor cell proportion in tissue samples for molecular testing improves interobserver reproducibility of its assessment.](https://www.ncbi.nlm.nih.gov/pubmed/27853865) Virchows Arch 470(1):21-27. |
| QuiP | “Qualitätssicherungs-Initiative Pathologie”/Quality assurance Initiative Pathology | quip.eu | - Petersen I, Dietel M, Geilenkeuser WJ, Mireskandari M, Weichert W, Steiger K, Scheel AH, Büttner R, Schirmacher P, Warth A, Lasitschka F, Schildhaus HU, Kirchner T, Reu S, Kreipe H, Länger F, Tiemann M, Schulte C, Jöhrens K (2017) [EGFR immunohistochemistry as biomarker for antibody-based therapy of squamous NSCLC - Experience from the first ring trial of the German Quality Assurance Initiative for Pathology (QuIP®).](https://www.ncbi.nlm.nih.gov/pubmed/29108919) Pathol Res Pract 213(12):1530-1535. - Fassunke J, Ihle MA, Lenze D, Lehmann A, Hummel M, Vollbrecht C, Penzel R, Volckmar AL, Stenzinger A, Endris V, Jung A, Lehmann U, Zeugner S, Baretton G, Kreipe H, Schirmacher P, Kirchner T, Dietel M, Büttner R, Merkelbach-Bruse S (2017) [EGFR T790M mutation testing of non-small cell lung cancer tissue and blood samples artificially spiked with circulating cell-free tumor DNA: results of a round robin trial.](https://www.ncbi.nlm.nih.gov/pubmed/28884371) Virchows Arch 471(4):509-520. |
| RCPAQAP | Royal College of Pathologists of Australasia Quality Assurance Programs | rcpaqap.com.au | Pagliuso S, Parry S, Haffajee Z, Badrick T, Miller K (2018) The challenges of implementing a PD-L1 proficiency testing program in Australia. Vascular Cell 10(1):2 |
| SEAP | “Sociedad Española de Anatomía Patológica”/ Spanish Society of Anatomic Pathology | www.seap.es/calidad |  |
| UK NEQAS for molecular genetics/ GenQA | United Kingdom National External Quality Assessment Service for Molecular Genetics/ Genomics External Quality Assessment | www.genqa.org | - Richman SD, Fairley J, Butler R, Deans ZC (2017) [RAS screening in colorectal cancer: a comprehensive analysis of the results from the UK NEQAS colorectal cancer external quality assurance schemes (2009-2016).](https://www.ncbi.nlm.nih.gov/pubmed/28653203) Virchows Arch 471(6):721-729. - Deans ZC, Wallace A, O'Sullivan B, Purvis A, Camus S, Fairley JA, Gonzalez D (2014) [External quality assessment of BRAF molecular analysis in melanoma.](https://www.ncbi.nlm.nih.gov/pubmed/24098023) J Clin Pathol 67(2):120-4. |
| UK NEQAS for ICC & ISH | United Kingdom National External Quality Assessment Service for Immunohistochemistry and In-Situ Hybridisation | www.ukneqasiccish.org | - Dodson A, Parry S, Ibrahim M, Bartlett JM, Pinder S, Dowsett M, Miller K (2018) Breast cancer biomarkers in clinical testing: analysis of a UK national external quality assessment scheme for immunocytochemistry and in situ hybridisation database containing results from 199 300 patients. J Pathol Clin Res 4(4):262-273. - Ibrahim M, Parry S, Wilkinson D, Bilbe N, Allen D, Forrest S, Maxwell P, O'Grady A, Starczynski J, Tanier P, Gosney J, Kerr K, Miller K, Thunnissen E (2016) ALK Immunohistochemistry in NSCLC: Discordant Staining Can Impact Patient Treatment Regimen. [J Thorac Oncol](https://www.ncbi.nlm.nih.gov/pubmed/27468935) 11(12):2241-2247. |
| cIQc | Canadian Immunohistochemistry Quality Control program | cpqa.ca | - Pérez T, Makrestsov N, Garatt J, Torlakovic E, Gilks CB, Mallett S (2016) Modeling Canadian Quality Control Test Program for Steroid Hormone Receptors in Breast Cancer: Diagnostic Accuracy Study. Appl Immunohistochem Mol Morphol 24:679–687. - Cheung CC, Garratt J, Won J, Cutz JC, Gilks BC, Tsao M, Torlakovic EE (2015) [Developing ALK Immunohistochemistry and In Situ Hybridization Proficiency Testing for Non-Small Cell Lung Cancer in Canada: Canadian Immunohistochemistry Quality Control Challenges and Successes.](https://www.ncbi.nlm.nih.gov/pubmed/26551338) Appl Immunohistochem Mol Morphol 23(10):677-81. |
| NordiQC | Nordic Immunohistochemical Quality Control | www.nordiqc.org | Vyberg M, Nielsen S (2016) Proficiency testing in immunohistochemistry-experiences from Nordic Immunohistochemical Quality Control (NordiQC). Virchows Arch 468(1):19-29. |

*Only data from providers who collaborated are shown

**Supplementary table 2** Tasks and criteria for competence of parties involved

| Involved party | Tasks | Criteria for competence |
| --- | --- | --- |
| Society (e.g. ESP) | - Manage the budget for organizing the EQA program - Install working parties for EQA |  |
| EQA coordinator | Elements from ISO/IEC 17043:2010 (4.2.4) [5]:   - select appropriate test items^§^ - plan EQA programs (timeline and design)* - perform sampling^§^ - facilitate measurements to determine stability and homogeneity, assigned values and associated uncertainties of the measurements or qualitative results of the test item^§^ - prepare, handle and distribute test items^§^ - operate the data processing system^§^ - conduct statistical analysis, if applicable^§^ - evaluate the performance of EQA participants* - give opinions and interpretations^§^ - authorize the issue of EQA reports*   Additional elements identified by the EQA providers:   - employ “fit-for-purpose” approach to the design of EQA program - select which TPCs are addressed by each EQA program - create assessor teams with appropriate expertise - engage statistical consultation when it is relevant (depending on the TPC evaluated) - coordinate an appeal system - monitor of laboratory performance over time by using selected (designated) laboratory monitoring parameters, tools for following trends of performance, and triggers for intervention when relevant - communicate results to regulatory bodies, if applicable - communicate EQA data in meetings/conferences | Elements from van Krieken et al. [41]:   - should have experience in quality management - should have a solid background in the diagnostic domain of the EQA program - should have the necessary facilities to run an EQA program   Additional elements identified by the EQA providers:   - should have a quality management system to facilitate corrective and preventive actions, risk reduction and continuous improvement - should know the workflow of laboratories - should have theoretical experience with and access to validated methods - should have means to access various types of professional consultants as and when needed (statistician, molecular biologist, oncologist, equipment company technical support, etc.) - should be members of the IQNPath or other professional organization that facilitates harmonization and standardization of EQA practices |
| Subject experts | Elements from ISO/IEC 17043:2010 (4.4.1.5) [5]°:   - provide input/consultation to EQA coordinator and other medical or technical experts - identification and resolution of any difficulties expected in the preparation and maintenance of homogeneous proficiency test items, or in the provision of a stable assigned value for a proficiency test item - advise on the preparation of detailed instructions for participants - comment on any technical difficulties or other remarks raised by participants in previous proficiency testing rounds - provision of advice to other medical or technical experts in evaluating the performance of participants - commenting on the results and performance of participants as a whole and, where appropriate, groups of participants or individual participants - responding to feedback from participants if appropriate, including appeals - advising or participating in technical meetings with participants   Additional elements identified by the EQA providers:   - approving the criteria for assessing laboratory performance - presenting data during symposia/conferences | Elements identified by the EQA providers:   - should have knowledge regarding medical and technical aspects of the methods used by the laboratories that participate in the EQA program - should be working in a diagnostic laboratory or an institute of pathology accredited according to ISO/IEC 15189 (or 17020) by the recognized national accreditation body or certified by a national cancer centre, if available (e.g. OncoZert, the certifying body of the German Cancer Society) [3, 4, 7] |
| Assessor | Elements identified by the EQA providers:   - advise on the development of evaluation criteria as set by the EQA coordinator, medical- and technical expert - evaluate participants’ results - comment on and score the results and performance of participants as a whole and, where appropriate, groups of participants or individual participants - give feedback on the summarizing report - suggest points for improvement | Elements from van Krieken et al. [41]:   - should have knowledge regarding medical and technical aspects of the methods used by the laboratories that participate in the EQA program   Elements identified by the EQA providers:   - should be involved in predictive testing for oncology in a diagnostic laboratory preferably accredited according to ISO/IEC 15189 (or 17020) by the recognized national accreditation body or certified by a national cancer centre, if available (e.g. OncoZert, the certifying body of the German Cancer Society) [3, 4, 7] - should have knowledge in EQA and quality assurance |
| Laboratory performing EQA sample preparations | Elements identified by the EQA providers   - (source) and prepare EQA samples, including sample labelling - may retest samples in case of discordances between EQA provider gold standard results and participant results | Elements identified by the EQA providers:   - accredited according to ISO/IEC 15189 (ISO/IEC 17043:2010 (4.1)), (or 17025)) for the tested parameter [3, 4, 7] - experience in routine testing of samples the test for which the EQA program is being set up - access to the samples (in accordance with national legal provisions) - ability to prepare samples according to instructions from the EQA provider - demonstrable high-quality testing - if relevant: promote the EQA program in your own country |
| IQNPath | - Harmonize practices between EQA providers - Exchange expertise between stakeholders | Elements identified by the EQA providers:   - experience in the field of biomarker testing in oncology - representative composition of the board |

*: activities that cannot be subcontracted

^§^: activities that can be subcontracted, but the coordinator remains responsible for selection and evaluation of the subcontractor
°: ISO/IEC 17043:2010 (4.4.1.5) only refers to ‘technical expertise’

**Supplementary Table 3** Examples of sample types used by different EQA providers

| EQA Provider* | Program Purpose | Examples | |
| --- | --- | --- | --- |
|  |  | Type | Reason for Inclusion |
| AIOM | EGFR EQA programs in NSCLC and EQA programs for colon and melanoma; evaluation of technical genotyping accuracy. | FFPE slides from patient material and/or artificial reference materials | Patient samples mimic daily routine.  Reference material mimics challenging small biopsies |
| AIOM, EMQN, ESP, UK NEQAS /GenQA (coordinated by IQNPath) | European pilot EQA program for ctDNA analysis; assessment of overall laboratory accuracy, including protocol-, interpretation- and reporting assessment. | Reference materials: cfDNA spike-in samples in artificial plasma or normal donor plasma | No patient material available for liquid biopsies |
| EMQN | Lung, colorectal and melanoma programs; evaluation of overall laboratory accuracy, including protocol-, interpretation and reporting | FFPE slides; 2 from patient material and 8 artificial reference materials | With many participants, it is too difficult to obtain sufficient patient material for 10 samples. Patient samples must be controlled for heterogeneity whilst reference materials can be used to obtain a wider range of mutations, and explore quoted LODs as all participants receive a homogeneous sample |
|  | Panel program; evaluation of technical genotyping accuracy (across a range of genes, including challenging VAFs) | FFPE slides: 3 artificial reference materials | Samples with multiplex mutations can challenge laboratories offering panel testing |
| ESP | ESP Lung EQA scheme and ESP Colon EQA scheme; evaluation of overall laboratory accuracy, staining quality (IHC), test outcome (IHC, FISH, DNA and mRNA) and reporting. | FFPE slides from patient material, occasionally supplemented by reference materials | Patient samples mimic daily routine Reference materials occasionally used to answer specific questions (e.g. multiple mutations in 1 sample) |
|  | ESP LUNG EQA scheme; evaluation of readout accuracy and result interpretation | Digital microscope slide images | Digital images represent actual samples for “readout” component of the analytical phase and ensure that all participants are sharing the same “tissue sample”, which is otherwise difficult due to tissue heterogeneity |
| QuiP | Evaluation of overall laboratory accuracy, including protocol, interpretation and reporting | FFPE slides from patient material  Blood with cell-line derived cells | Patient samples mimic daily routine.  No patient material available for liquid biopsies |
| RCPAQAP | IHC programs; evaluation of overall laboratory accuracy, including protocol- and interpretation | FFPE slides from patient material | Patient samples mimic daily routine. |
|  | HER2 BRISH Breast Diagnostic | Digital microscope slide images | Cost-effective and homogeneous samples |
| UK NEQAS for Molecular Genetics/GenQA | Lung cancer EQA; Additional lung biomarkers;  Colorectal cancer EQA; Melanoma EQA;  Gastrointestinal stromal tumours EQA;  Microsatellite instability EQA; Mismatch repair (*BRAF* mutations and *MLH1* promoter methylation) EQA;  Molecular Tissue identification EQA;  Sarcoma EQA;  CNS tumours EQA  Assessment of overall laboratory accuracy, including protocol-, interpretation- and reporting assessment | FFPE slides and/or rolled sections from patient material | Patient samples mimic daily routine. |
|  | DNA extraction from FFPE tissue EQA. Technical assessment of DNA quantity and quality | FFPE slides and/or rolled sections from patient material | Patient samples mimic daily routine. |
|  | Tissue-i – assessment of individual’s ability to determine tumour content/cellularity of samples for molecular analysis | Digital microscope slide images | Digital images represent actual samples for “readout” component of the analytical phase and ensure that all participants are sharing the same “tissue sample”, which is otherwise difficult due to tissue heterogeneity. |
|  | DNA extraction from FFPE tissue EQA. Technical assessment of DNA quantity and quality | Fresh tissue | Patient samples mimic daily routine. |

*Only data from providers who collaborated are shown, FFPE: formalin-fixed paraffin embedded, ctDNA: circulating tumour DNA, cfDNA: cell free DNA, *EGFR*: epidermal growth factor receptor, NSCLC: non-small cell lung cancer, HER2: human epidermal growth factor receptor 2, BRISH: bright field in situ hybridization

**Supplementary Table 4** Overview of scoring systems for the evaluation of IHC staining quality

| EQA Provider* | System | Grade meaning |
| --- | --- | --- |
| ESP & UK NEQAS for ICC & ISH | Numerical grading | 1: failure to stain the slides and no interpretation possible 2: staining is incorrect and clinical output affected 3: staining is incorrect but clinical output not affected 4: very good staining with minor remark 5: excellent staining  Threshold for successful participation: a minimum score of 3/5 |
| cIQc | Pass or Fail for predictive IHC biomarkers  Qualitative grading for diagnostic biomarkers | “Diagnostic sensitivity and specificity acceptable” or “Diagnostic sensitivity and specificity not acceptable” **  Poor, borderline, good, optimal (or modification of this when necessary based on the purpose of the PT run/challenge)  Threshold for successful participation: scheme dependent |
| NordiQC | Qualitative grading | Poor Borderline Good Optimal  Threshold for successful participation: good or optimal staining |
| RCPAQAP | Numerical grading | 0: no staining or incorrect antibody applied 1: very poor staining and not diagnosable 2: unsatisfactory staining and diagnosis would be affected 3: staining criteria have been met at basic level  4: above average staining 5: perfect fulfilment of criteria  Threshold for successful participation: a minimum score of 3/5 |

*Only data from providers who collaborated are shown
** Note: Defining the level of what represent acceptable diagnostic accuracy is not straightforward and should be done using guidelines (e.g. from ASCO/CAP for breast cancer biomarkers ER, PR, or HER2), although not available yet for all biomarkers (e.g. PD-L1) [30, 44]. However, 90% cut off point is used for most tests by cIQc. AIOM, EMQN, Gen&Tiss, Quip and SEAP not represented as no schemes for IHC assessment are organized.

**Supplementary Table 5** Example of reporting elements for assessment and the required mock information

| Element | Provision of mock information* | ESP | Gen&Tiss | UK NEQAS for Molecular Genetics/ GenQA |
| --- | --- | --- | --- | --- |
| Result interpretation | Patient history in request form | Points given | Points given | Points given |
| Patient name | Patient name in request form | Points given | Points given | Points given |
| Date of birth | Date of birth in request form | Points given | Points given | Points given |
| Reason for testing | Reason for referral in request form | Comment given | Points given | Comment given |
| Sample number | Present on sample | Comment given | Points given | Points given |
| Sample nature | Sample nature in request form | Comment given | Points given | Comment given |
| Date of sample collection | Date of sample collection in request form | Score given |  | Points given |
| Patient history | Patient history in request form | Comment given | Points given | Comment given |
| Neoplastic cell content/ DNA concentration | Output of the testing process | Comment given | Points given | Points given |
| Method description | Known by the laboratory | Points given | Points given | Points given |
| Test limitations  (alterations tested, testing sensitivity) | Known by the laboratory | Points given | Points given | Points given |
| Reference sequence | Know by the laboratory | Points given |  | Points given |
| Report layout and content - page number and total number of pages - report title - name and address test requester  - date of report validation - name of report validator | Standard report layout of the laboratory | - Page number and total number of pages: comment given - Other elements: not assessed | - Unique identifier: points given - Page number and total number of pages:   points given   - Report title: points given - Name and address requester: points given - Date of sample collection: points given - Sample arrival date: points given - Report date: points given - Name of pathologist: points given | - Three unique identifiers: points given - Page number and total number of pages:   points given   - Report title: comment given - Name and address requester: comment given - Date of sample collection: points given - Sample arrival date: points given - Report date: points given   indication of authorizer: comment given |

* Should reflect the current, routine situation in healthcare.

**Supplementary Table 6** Criteria for defining successful participation

| EQA provider* | Criteria |
| --- | --- |
| AIOM | Score ≥ 18 out of 20 points and no critical errors in genotyping  *Critical errors in genotyping include false positive and false negative results* |
| EMQN | No critical errors in genotyping or interpretation  *Critical errors in genotyping include anything which can be deemed to potentially cause patient harm (e.g. false positive or negative result) Critical errors in interpretation include anything which can be deemed to potentially cause patient harm such as recommending the prescription of a drug when the patient does not have a mutation in a relevant gene* |
| ESP Colon EQA scheme | Test outcome: score ≥ 90% and no genotyping error  *Genotyping errors include false positives, false negatives and the identification of the wrong mutation* |
| ESP Lung EQA scheme | Test outcome: score ≥ 90% and no genotyping error  Reporting: score ≥ 90%  *Genotyping errors include false positives, false negatives and the identification of the wrong mutation* |
| Gen&Tiss | Test outcome: score ≥ 90% |
| QuiP | Score ≥ 90%, mostly ≥ 95% |
| UK NEQAS for Molecular Genetics/GenQA | No critical errors in genotyping or interpretation  *Critical errors in genotyping include anything which can be deemed to potentially cause patient harm (e.g. false positive or negative result). Critical errors in interpretation include anything which can be deemed to potentially cause patient harm such as recommending the prescription of a drug when the patient does not have a mutation in a relevant gene.* |

**Only data from providers who collaborated are shown*
