# Supplementary material for: Biomarker testing in oncology – Requirements for organizing external quality assessment programs to improve the performance of laboratory testing: revision of an expert opinion paper on behalf of IQNPath ABSL
Source: Virchows Arch. 2020 Oct 13;478(3):553–65. doi: 10.1007/s00428-020-02928-z (PMC7550230; doi:10.1007/s00428-020-02928-z)
Supplement: Supplementary file 2 — (DOCX 47 kb) [file 428_2020_2928_MOESM2_ESM.docx]

Supplementary Data File 1– survey overview of survey questions and results

Q1: Which schemes do you provide?

| EQA provider | Molecular schemes | IHC schemes | FISH schemes |
| --- | --- | --- | --- |
| AiOM | Yes |  |  |
| EMQN | Yes |  |  |
| ESP | Yes | Yes |  |
| GenQA | Yes |  |  |
| Gen&Tiss | Yes |  |  |
| QuIP | Yes | Yes | Yes |
| SEAP | Yes | Yes |  |
| cIQC |  |  |  |
| NordiQC |  | Yes | Yes |
| UKNEQAS for ICC/ISH |  |  |  |

***Part 1: molecular EQA schemes***

Q2: Are you accredited/certified as an EQA provider? By which accreditation office?

| EQA provider | Accredited/certified (standard) | Accreditation office |
| --- | --- | --- |
| AiOM | No |  |
| EMQN | Yes (ISO 17043) | UKAS |
| ESP | Yes (ISO 17043) | BELAC |
| GenQA | Yes (ISO 17043) | UKAS |
| Gen&Tiss | Yes (ISO 17043 and ISO9001) | BELAC and Veritas |
| QuIP | In preparation |  |
| SEAP | In preparation |  |
| cIQC |  |  |
| NordiQC | No |  |
| UKNEQAS for ICC/ISH | Yes (ISO 17043) | UKAS |

Q3: Which experts are included in the organization of molecular EQA schemes your EQA organization provides?

| EQA provider | Medical expert | Technical expert | Representative EQA provider |
| --- | --- | --- | --- |
| AiOM | Yes (pathologist + oncologist) | Yes | Yes |
| EMQN | Yes (pathologist + oncologist) | Yes | Yes |
| ESP | Yes (pathologist) | Yes | Yes |
| GenQA | Yes (pathologist) | Yes | Yes |
| Gen&Tiss | Yes (oncologist) | Yes | Yes |
| QuIP | Yes (pathologist + biologist) | Yes | Yes |
| SEAP | Yes (oncologist) | Yes | Yes |

Q4: Which parts are covered by the molecular EQA schemes your EQA organization provides?

| EQA provider | Pathology review | Test result | Reporting |
| --- | --- | --- | --- |
| AiOM | No | Yes | No |
| EMQN | No | Yes | Yes |
| ESP | No | Yes | Yes |
| GenQA | Yes | Yes | Yes |
| Gen&Tiss | Yes | Yes | Yes |
| QuIP | Yes | Yes | Yes |
| SEAP | Yes | Yes | Yes |

Q5: How many weeks is the turnaround time that your organization sets for its participants?

| EQA provider | TAT |
| --- | --- |
| AiOM | 3 weeks (10 samples) or 4 weeks (14 samples) |
| EMQN | 8 weeks |
| ESP | 2 weeks |
| GenQA | 6 weeks |
| Gen&Tiss | 3 weeks |
| QuIP | 2 weeks |
| SEAP | 4 weeks |

Q6: How many samples are being distributed per scheme? Number of runs? Number of samples per run?

| EQA provider | N of samples per scheme | N of runs per scheme | N of samples per run |
| --- | --- | --- | --- |
| AiOM | 10 | 1 | 10 |
| EMQN | 10 | 1 | 10 |
| ESP | 10 | 3 | 3 or 4 |
| GenQA | 3 or 9 | 1 or 2 | 3 or 4 or 5 |
| Gen&Tiss | 5/tissue | 1 | 5 |
| QuIP | 10 | 1 | 10 |
| SEAP | 8 | 2 | 4 |

Q7: Which sample types are distributed for the molecular EQA schemes your EQA organization provides?

| EQA provider | Patient FFPE | Cell line FFPE | Cell line | Commercial sample | Digital case | Plasma |
| --- | --- | --- | --- | --- | --- | --- |
| AiOM | Yes | Yes | No | Yes | No | Yes |
| EMQN | Yes | Yes | No | Yes | No | Yes |
| ESP | Yes | Yes | No | No | Yes | Yes |
| GenQA | Yes | Yes | No | Yes | No | Yes |
| Gen&Tiss | Yes | No | No | Yes | No | Yes |
| QuIP | Yes | No | No | No | No | Yes |
| SEAP | Yes | No | No | No | No | No |

Q8: To which extent do you feel that using only FFPE samples from patients is practically feasible?

| EQA provider | Feasibility |
| --- | --- |
| AiOM | Neutral |
| EMQN | Not feasible |
| ESP | Not feasible |
| GenQA | Feasible |
| Gen&Tiss | Not feasible |
| QuIP | Feasible |
| SEAP | Neutral |

Q9: Do you use ‘challenging samples’ in addition to common samples?

| Use of challenging samples | AiOM | EMQN | ESP | GenQA | Gen&Tiss | QuIP | SEAP |
| --- | --- | --- | --- | --- | --- | --- | --- |
| Yes and taken into account for successful participation | Yes | Yes |  |  |  | Yes | Yes |
| Yes, but not taken into account for successful participation |  |  |  | Yes | Yes |  |  |
| No |  |  | Yes |  |  |  |  |

Q10: What do you consider as ‘challenging samples’?

|  | AiOM | EMQN | ESP | GenQA | Gen&Tiss | QuIP | SEAP |
| --- | --- | --- | --- | --- | --- | --- | --- |
| Neoplastic cell percentage around diagnostic cut-off of method | Yes | Yes | Yes | Yes | Yes | Yes | Yes |
| Contamination with immune cells, necrosis, desmoplastic stroma | Yes | Yes | Yes | Yes |  |  | Yes |
| Low VAF | Yes | Yes | Yes | Yes |  |  | Yes |
| Low DNA quality | Yes | Yes | Yes | Yes |  |  | Yes |
| Rare mutation |  |  |  |  | Yes |  |  |

Q11: Are the following criteria for reference laboratories fulfilled in your organisation?
     a. The reference laboratory should be a fully equipped molecular pathology laboratory with certified pathologists, clinical molecular biologists and
 technicians.
     b. The laboratory should be accredited to a recognized international standard (e.g. ISO 15189)
     c. The laboratory should have passed an EQA test

| EQA provider | Criteria met | Number of reference laboratories |
| --- | --- | --- |
| AiOM | Yes | 3 |
| EMQN | Yes | 4 |
| ESP | Yes | 2 |
| GenQA | Yes | 2 |
| Gen&Tiss | Yes | 2 |
| QuIP | Yes | 3 |
| SEAP | Yes | 2 |

Q12: Which aspects are validated (pathology review, DNA quality, DNA quantity)?

|  | AiOM | EMQN | ESP | GenQA | Gen&Tiss | QuIP | SEAP |
| --- | --- | --- | --- | --- | --- | --- | --- |
| Pathology review: % neoplastic cells | Yes | Yes | Yes | Yes | Yes | Yes | Yes |
| DNA quality | Yes | Yes | Yes | Yes | Yes | Yes | Yes |
| DNA quantity | Yes | Yes | Yes | Yes | Yes | Yes | Yes |

Q13: Are the results assessed independently by at least 2 members of the assessment team (professionals with experience in the field of diagnostic molecular pathology), by comparing them with validated results and using predefined criteria. And are results subsequently discussed during an assessment meeting?

| EQA provider | Criteria met |
| --- | --- |
| AiOM | Yes |
| EMQN | Yes |
| ESP | Yes |
| GenQA | Yes |
| Gen&Tiss | Yes |
| QuIP | Yes |
| SEAP | Yes |

Q14: Which elements are marked in the pre-analytical phase? (adequacy of the sample, neoplastic cell percentage determination, evaluation of the need for dissection)

|  | AiOM | EMQN | ESP | GenQA | Gen&Tiss | QuIP | SEAP |
| --- | --- | --- | --- | --- | --- | --- | --- |
| Sample adequacy | Not taken into account for final score | Not taken into account for final score | Taken into account for final score | Not taken into account for final score | Not taken into account for final score | Taken into account for final score | Taken into account for final score |
| Neoplastic cell % determination | Not taken into account for final score | Not taken into account for final score | Not taken into account for final score | Not taken into account for final score | Not taken into account for final score | Taken into account for final score | Taken into account for final score |
| Need for dissection | Not taken into account for final score | Not applicable: only scrolls provided | Not taken into account for final score | Not taken into account for final score | Not taken into account for final score | Taken into account for final score | Taken into account for final score |

Q15: Is the genotyping result evaluated from a datasheet or uploaded reports and based on which scoring criteria?

| EQA provider | Results evaluated on | Scoring criteria based on |
| --- | --- | --- |
| AiOM | Yes | Guideline by van Krieken et al. |
| EMQN | Yes | Guideline by van Krieken et al. + self-developed criteria |
| ESP | Yes | Guideline by van Krieken et al. |
| GenQA | Yes | Guideline by van Krieken et al. + self-developed criteria |
| Gen&Tiss | Yes | Guideline by van Krieken et al. + self-developed criteria |
| QuIP | Yes | Guideline by van Krieken et al. + self-developed criteria |
| SEAP | Yes | Self-developed criteria |

Q16: Are points deducted when the participant is not able to identify the exact alteration because the kit is not able to?

| EQA provider | Results evaluated on |
| --- | --- |
| AiOM | No |
| EMQN | No |
| ESP | Yes |
| GenQA | No |
| Gen&Tiss | Yes |
| QuIP | Yes |
| SEAP | No |

Q17: Are points deducted when there is no result for a certain gene because of cascade testing (e.g. no result for EGFR because of a mutation in KRAS)?

| EQA provider | Results evaluated on |
| --- | --- |
| AiOM | No |
| EMQN | No |
| ESP | Yes |
| GenQA | No |
| Gen&Tiss | Yes |
| QuIP | No |
| SEAP | No |

Q18: What is according to you the minimal threshold above which laboratories should be able to detect aberrations? (e.g. a variant allelic frequency of 5%)

| EQA provider | Threshold (VAF) |
| --- | --- |
| AiOM | Depends on clinical relevance |
| EMQN | Method dependent |
| ESP | 10% |
| GenQA | Method dependent |
| Gen&Tiss | 5% |
| QuIP | Depends on clinical relevance |
| SEAP | Depends on clinical relevance |

Q19:  Do you deduct points when the laboratory is not able to detect an aberration with a method that has an analytic sensitivity above this threshold?

| EQA provider | Threshold (VAF) |
| --- | --- |
| AiOM | Yes |
| EMQN | Method dependent |
| ESP | Method dependent |
| GenQA | Method dependent |
| Gen&Tiss | Method dependent |
| QuIP | Depends on clinical relevance |
| SEAP | Depends on clinical relevance |

Q20: According to the HGVS guidelines, old nomenclature should not be used anymore. Do you deduct points if laboratories still use this?

| EQA provider | Results evaluated on |
| --- | --- |
| AiOM | Yes, but not if the new nomenclature is used in addition to the old nomenclature |
| EMQN | Yes, but not if the new nomenclature is used in addition to the old nomenclature |
| ESP | Yes, also in case the alteration is reported multiple times and the old and new nomenclature are both used |
| GenQA | Yes, but not if the new nomenclature is used in addition to the old nomenclature |
| Gen&Tiss | Yes, also in case the alteration is reported multiple times and the old and new nomenclature are both used |
| QuIP | No |
| SEAP | No |

Q21: Are reports evaluated? How many are evaluated?

| EQA provider | Results evaluated on | Number |
| --- | --- | --- |
| AiOM | No |  |
| EMQN | Yes and taken into account for final score | 2 or 3 |
| ESP | Yes, but not taken into account for final score | 3 |
| GenQA | Yes and taken into account for final score | All |
| Gen&Tiss | Yes, but not taken into account for final score | 3 |
| QuIP | No |  |
| SEAP | No |  |

Q22: Which items are marked on the reports?

|  | EMQN | ESP | GenQA | Gen&Tiss |
| --- | --- | --- | --- | --- |
| Incorrect name and first name | -1 pt | -1 pt | -1 pt | -1 pt |
| Incorrect date of birth | -1 pt | -1 pt | ND | -1 pt |
| No results and explanation | 1.0 | -0,75 pt | -1 or -0,5 pt | -1 pt |
| No clinical interpretation | -1.5 | -0,5 pt | -1 or -0,5 pt | -1 pt |
| No reference sequence and version | 0.2 | -0,25 pt | -0,5 pt | -1 pt |
| No specification of assay and regions | 0.5 | -0,125 pt | -0,5 pt | -1 pt |
| No test limitations/sensitivity | 0.2 | -0,25 pt | -0,5 pt | -1 pt |
| No neoplastic cell percentage | 0.5 | Comment given | Comment given | -1 pt |
| Patient history missing | Comment given | Comment given | ND | -1 pt |
| Length > 1 page | Comment given | ND | ND | Comment given |
| Spelling error | Comment given | Comment given | ND | Comment given |
| Report authorizer absent | Comment given | Comment given | ND | -1 pt |
| No clear presentation of results | Comment given | Comment given | Comment given | ND |
| No name/address of referring person | Comment given | ND | Comment given | ND |

ND: no deduction

Q23: Is 'too direct patient advice' assessed by your EQA organization?

| EQA provider | Type of interpretation asked | Assessed? |
| --- | --- | --- |
| EMQN | Patient specific | Yes |
| ESP | General | Yes |
| GenQA | Patient specific | Yes |
| Gen&Tiss | General | Yes |

Q24: Which of the following characteristics of testing methods are assessed on the report (full kit name, supplier, version/lot number, detection platform)

| EQA provider | Full kit name | Kit supplier | Kit version/lot number | Detection platform |
| --- | --- | --- | --- | --- |
| EMQN | Yes | Yes | Yes | Yes |
| ESP | Yes | Yes |  | Yes |
| GenQA | Yes | Yes | Yes |  |
| Gen&Tiss | Yes | Yes |  | Yes |

Q25: Which parts of the assessment does your EQA organization take into account to decide whether the laboratory is successful or not?

|  | AiOM | EMQN | ESP | GenQA | Gen&Tiss | QuIP | SEAP |
| --- | --- | --- | --- | --- | --- | --- | --- |
| Genotyping | Yes | Yes | Yes | Yes | Yes | Yes | Yes |
| Interpretation |  | Yes |  | Yes |  |  | Yes |

Q26: What do you set as a cut-off value for successful participation (e.g. a score of 90%)?

| EQA provider | Cut-off |
| --- | --- |
| AiOM | 90% and absence of major genotyping errors (FN/FP) |
| EMQN | Absence of critical genotyping and interpretation errors |
| ESP | 90% |
| GenQA | Absence of critical genotyping and interpretation errors |
| Gen&Tiss | 80% |
| QuIP | 90% for Liquid biopsy, 95% for other schemes and 100% for schemes with only 5 samples |
| SEAP | 75% |

Q27: How does your EQA organization act upon participants that participate to validate/verify their test methods?

|  | AiOM | EMQN | ESP | GenQA | Gen&Tiss | QuIP | SEAP |
| --- | --- | --- | --- | --- | --- | --- | --- |
| Included in average score of the EQA scheme | Yes | Yes | Yes | Yes | Yes | Yes |  |
| Not included in average score of the EQA scheme |  |  |  |  |  |  | Yes |

Q28: What actions are taken regarding poor performers? (poor performer = unsuccessful participation during 1 scheme participation)

|  | AiOM | EMQN | ESP | GenQA | Gen&Tiss | QuIP | SEAP |
| --- | --- | --- | --- | --- | --- | --- | --- |
| Laboratories are individually encouraged to improve their practices | Yes |  | Yes |  | Yes |  | Yes |
| Laboratories should provide an improvement plan to the EQA provider |  | Yes |  | Yes |  |  |  |
| Regulatory bodies are notified |  | Yes (UK, Switzerland, Australia) |  | Yes (UK, Switzerland, Australia) |  |  |  |

Q29: What actions are taken regarding persistent poor performers?

|  | AiOM | EMQN | ESP | GenQA | Gen&Tiss | QuIP | SEAP |
| --- | --- | --- | --- | --- | --- | --- | --- |
| Laboratories are individually encouraged to improve their practices | Yes |  | Yes |  | Yes |  | Yes |
| Laboratories should provide an improvement plan to the EQA provider |  | Yes |  | Yes |  |  |  |
| Regulatory bodies are notified |  | Yes (UK, Switzerland, Australia) |  | Yes (UK, Switzerland, Australia) |  |  |  |

Q30: Is it the responsibility of the EQA provider to take actions against a (persistent) poor performer?

|  | AiOM | EMQN | ESP | GenQA | Gen&Tiss | QuIP | SEAP |
| --- | --- | --- | --- | --- | --- | --- | --- |
| Poor performer | No | No | Yes | Yes | No | No | No |
| Persistent poor performer | No | Yes | Yes | Yes | No | No | No |

Q31: Does your EQA provider organization give participants prior notice before sending EQA samples?

|  | AiOM | EMQN | ESP | GenQA | Gen&Tiss | QuIP | SEAP |
| --- | --- | --- | --- | --- | --- | --- | --- |
| Yes, in the form of a participants’ manual | Yes | Yes |  | Yes | Yes |  | Yes |
| Yes, in the form of an email | Yes | Yes | Yes | Yes |  | Yes | Yes |
| Yes, in the form of an announcement on the website |  | Yes | Yes | Yes |  | Yes | Yes |
| Yes, via social media |  | Yes |  | Yes |  |  |  |

Q32: Which elements are included in the participants’ manual?

|  | AiOM | EMQN | ESP | GenQA | Gen&Tiss | QuIP | SEAP |
| --- | --- | --- | --- | --- | --- | --- | --- |
| General info about the EQA scheme | Yes | Yes | Yes | Yes | Yes | Yes | Yes |
| EQA processes and practical details | Yes | Yes | Yes | Yes | Yes | Yes | Yes |
| Terms and conditions | Yes | Yes | Yes | Yes | Yes | Yes | Yes |
| Details of how to apply | Yes | Yes | Yes | Yes | Yes | Yes | Yes |

Q33: Which of the following items are communicated to the laboratories?

|  | AiOM | EMQN | ESP | GenQA | Gen&Tiss | QuIP | SEAP |
| --- | --- | --- | --- | --- | --- | --- | --- |
| Information about fixation |  |  | Yes | Yes | Yes | Yes | Yes |
| Information about integrity |  | Yes | Yes | Yes | Yes | Yes | Yes |
| Clinical information/test request | Yes | Yes | Yes | Yes | Yes |  | Yes |
| Standardized questionnaire |  | Yes | Yes |  | Yes | Yes | Yes |

Q34: Are the correct genotyping outcomes available before the general report is sent out?

| EQA provider | Results available before general report |
| --- | --- |
| AiOM | No |
| EMQN | Yes 1 week after submission closes |
| ESP | Yes |
| GenQA | No |
| Gen&Tiss | Yes |
| QuIP | Yes |
| SEAP | Yes |

Q35: Which elements are present in the general report?

|  | AiOM | EMQN | ESP | GenQA | Gen&Tiss | QuIP | SEAP |
| --- | --- | --- | --- | --- | --- | --- | --- |
| Name and contact details of the EQA provider | Yes | Yes | Yes | Yes |  | Yes | Yes |
| Name and contact details of the program coordinator |  | Yes | Yes | Yes |  | Yes | Yes |
| Name(s), function(s) and signature(s) of person(s) authorizing the report |  | Yes | Yes | Yes |  | Yes | Yes |
| An indication of which activities are subcontracted |  | Yes | Yes | Yes |  | Yes | Yes |
| The date of issue and status of the report |  | Yes | Yes | Yes |  | Yes | Yes |
| Page numbers and a clear indication of the end of the report |  | Yes | Yes | Yes |  | Yes |  |
| A statement on the extent to which results are confidential |  | Yes | Yes | Yes |  | Yes |  |
| The report number and clear identification of the EQA program | Yes | Yes | Yes | Yes |  | Yes | Yes |
| A clear description of the EQA items used | Yes | Yes | Yes | Yes |  | Yes | Yes |
| Description of the preparation and homogeneity and stability of samples | Yes | Yes | Yes | Yes |  | Yes | Yes |
| Participant's results (individual and/or aggregate group results) | Yes | Yes | Yes | Yes |  | Yes | Yes |
| Statistical data and summaries (assigned values, acceptable ranges, graphs) | Yes | Yes | Yes | Yes |  | Yes | Yes |
| Procedures used to establish any assigned value | Yes | Yes | Yes | Yes |  | Yes | Yes |
| Details of the metrological traceability and measurement of uncertainty |  | Yes | Yes | Yes |  | Yes | Yes |
| Procedures used to establish the standard deviation for proficiency assessment or other criteria for evaluation | Yes | Yes | Yes | Yes |  |  | Yes |
| Assigned values and summary statistics for test methods/procedures used by each group of participants |  | Yes | Yes | Yes |  | Yes | Yes |
| Comments on participant's performance by the EQA provider and technical advisers |  | Yes | Yes | Yes |  | Upon request | Yes |
| Information about the design and implementation of the EQA program | Yes | Yes | Yes | Yes | Yes | Yes | Yes |
| Procedures used to statistically analyse the data |  | Yes | Yes | Yes |  | Yes | Yes |
| Advice on the interpretation of the statistical analysis |  | Yes | Yes | Yes |  | Yes | Yes |
| Comments or recommendations based on the outcomes of the EQA program |  | Yes | Yes | Yes |  | Yes | Yes |
| Details and identity of the cell lines and results of reference laboratories |  |  |  | Yes |  | Yes |  |
| Details of patient specimens and results of reference laboratories |  | Yes | Yes | Yes |  | Yes | Yes |
| Variation within and between participants and comparisons with any previous EQA rounds, similar proficiency testing programs of published precision data |  | Yes |  | Yes | Yes | Yes |  |
| Variation between methods or procedures |  | Yes |  | Yes | Yes | Yes |  |
| Possible sources of error and suggestions for improving performance |  | Yes | Yes | Yes | Yes | Yes |  |
| Advice and educational feedback as part of continuous improvement |  | Yes | Yes | Yes |  | Upon request | Yes |
| Situations where unusual factors make evaluation of results and comments on performance impossible |  | Yes |  | Yes |  | If necessary |  |
| Any other suggestions, recommendations or general comments | Yes | Yes | Yes | Yes | Yes | Yes |  |
| Conclusions |  | Yes |  | Yes | Yes | Yes |  |

***Part 2: IHC/ISH schemes***

Q1: How many working days is the turnaround time that your organization sets for its participants?

| EQA provider | TAT |
| --- | --- |
| ESP | 2 weeks |
| NordiQC | Not assessed |
| QuIP | 2 weeks |
| SEAP | Not assessed |

Q2: Which sample types are distributed?

|  | ESP | NordiQC | QuIP | SEAP |
| --- | --- | --- | --- | --- |
| FFPE from patients | Yes | Yes | Yes | Yes |
| FFPE from cell lines | Yes | Yes | If necessary |  |
| Cell lines |  |  |  |  |
| Commercial samples |  | Yes | As controls | Yes |
| Digital cases | Yes |  | Yes |  |

Q3: Are challenging samples included?

|  | ESP | NordiQC | QuIP | SEAP |
| --- | --- | --- | --- | --- |
| Yes, and taken into account for success |  | Yes | Yes | Yes |
| Yes, but not taken into account for success |  |  |  |  |
| No | Yes |  |  |  |

Q4: What is considered a ‘challenging sample’?

|  | ESP | NordiQC | QuIP | SEAP |
| --- | --- | --- | --- | --- |
| Contaminated with immune cells, necrosis, desmoplastic stroma, … | Yes | Yes |  | Yes |
| Borderline staining | Yes | Yes | Yes | Yes |

Q5: Which aspects are validated in the samples for IHC?

|  | ESP | NordiQC | QuIP | SEAP |
| --- | --- | --- | --- | --- |
| Pathology review: % neoplastic cells | Yes | Yes | Yes | Yes |
| Quality staining | Yes | Yes | Yes | Yes |
| DNA quantity | Yes | Yes | Yes | Yes |

Q6: Which aspects are validated in the samples for FISH?

|  | ESP | NordiQC | QuIP | SEAP |
| --- | --- | --- | --- | --- |
| Pathology review: % neoplastic cells | Yes | Yes | Yes | Yes |
| Quality staining | Yes | Yes | Yes | Yes |
| DNA quantity | Yes | Yes | Yes | Yes |

Q7: Which elements of the pre-analytical phase are assessed?

|  | ESP | NordiQC | QuIP | SEAP |
| --- | --- | --- | --- | --- |
| Sample adequacy | Taken into account for final score | Not assessed | Taken into account for final score | Taken into account for final score |
| Neoplastic cell % determination | Not taken into account for final score | Not assessed | Taken into account for final score | Taken into account for final score |
| Need for dissection | Not taken into account for final score | Not assessed | Taken into account for final score | Taken into account for final score |

Q8: Which elements are assessed in the analytical phase?

|  | ESP | NordiQC | QuIP | SEAP |
| --- | --- | --- | --- | --- |
| Assessment of results | Taken into account for final score | Taken into account for final score | Taken into account for final score | Taken into account for final score |
| Technical assessment | Taken into account for final score | Taken into account for final score | Taken into account for final score | Taken into account for final score |

Q9: Which test characteristics are assessed in the post-analytical phase for FISH?

|  | ESP | NordiQC | QuIP | SEAP |
| --- | --- | --- | --- | --- |
| Method description | Yes | No reports | Yes | Yes |
| Full kit name | Yes | No reports | Yes | Yes |
| Version/lot number | Yes | No reports | Yes | Yes |
| Total number of counted nuclei | Yes | No reports | Yes | Yes |
| Number of split signals | Yes | No reports | Yes | Yes |

Q10: Which test characteristics are assessed in the post-analytical phase for IHC?

|  | ESP | NordiQC | QuIP | SEAP |
| --- | --- | --- | --- | --- |
| Antibody clone | Yes | No reports | Yes | Yes |
| Antibody supplier | Yes | No reports | Yes | Yes |
| Detection system | Yes | No reports | Yes | Yes |

Q11: Which parts are taken into account for successful participation?

|  | ESP | NordiQC | QuIP | SEAP |
| --- | --- | --- | --- | --- |
| Pre-analytical phase | Yes | Yes |  | Yes |
| Analytical phase: identification of alteration | Yes | Yes | Yes | Yes |
| Analytical phase: technical assessment | Yes | Yes | Yes | Yes |
| Post-analytical phase | Yes |  |  | Yes |

Q12: What is the threshold for successful participation?

| EQA provider | TAT |
| --- | --- |
| ESP | 90% |
| NordiQC | Optimal/good staining |
| QuIP | 90% |
| SEAP | 75% |

Q13: Which actions are taken against poor performers?

|  | ESP | NordiQC | QuIP | SEAP |
| --- | --- | --- | --- | --- |
| Laboratories are individually encouraged to improve their practices | Yes | Yes |  | Yes |
| Laboratories should provide an improvement plan to the EQA provider |  |  |  |  |
| Regulatory bodies are notified |  |  |  |  |

Q14: Which actions are taken against persistent poor performers?

|  | ESP | NordiQC | QuIP | SEAP |
| --- | --- | --- | --- | --- |
| Laboratories are individually encouraged to improve their practices | Yes | Yes |  | Yes |
| Laboratories should provide an improvement plan to the EQA provider |  |  |  |  |
| Regulatory bodies are notified |  |  |  |  |
